# Supplementary material for: Prognostic value of 11C-methionine volume-based PET parameters in IDH wild type glioblastoma
Source: PLoS One. 2022 Feb 25;17(2):e0264387. doi: 10.1371/journal.pone.0264387 (PMC8880430; doi:10.1371/journal.pone.0264387)
Supplement: S1 File — (PDF) [file pone.0264387.s001.pdf]

Supplement 1

S1a – Paired analysis between MTV and clinical parameters

MTV and age

Variables in the Equation

	B	SE	Wald	df	Sig.	Exp(B)	95,0% CI for Exp(B)	
							Lower	Upper
MTV	,143	,056	6,536	1	,011	1,153	1,034	1,287
Age	-,076	,054	1,987	1	,159	,927	,834	1,030

MTV and extent of resection (EOR)

Variables in the Equation

	B	SE	Wald	df	Sig.	Exp(B)	95,0% CI for Exp(B)	
							Lower	Upper
MTV	,120	,048	6,230	1	,013	1,128	1,026	1,240
EOR	,264	,567	,216	1	,642	1,302	,428	3,958

MTV and radiotherapy dose

Variables in the Equation

	B	SE	Wald	df	Sig.	Exp(B)	95,0% CI for Exp(B)	
							Lower	Upper
MTV	,172	,065	7,035	1	,008	1,187	1,046	1,348
RTdose	,032	,026	1,533	1	,216	1,033	,981	1,087

S1b – Paired analysis between TLMM and clinical parameters

TLMM and age

Variables in the Equation

	B	SE	Wald	df	Sig.	Exp(B)	95,0% CI for Exp(B)	
							Lower	Upper
TLMM	,025	,011	4,989	1	,026	1,025	1,003	1,047
Age	-,083	,049	2,895	1	,089	,921	,837	1,013

TLMM and extent of resection (EOR)

Variables in the Equation

	B	SE	Wald	df	Sig.	Exp(B)	95,0% CI for Exp(B)	
							Lower	Upper
TLMM	,020	,010	3,908	1	,048	1,020	1,000	1,041
EOR	,554	,548	1,020	1	,312	1,740	,594	5,098

TLMM and radiotherapy dose

Variables in the Equation

	B	SE	Wald	df	Sig.	Exp(B)	95,0% CI for Exp(B)	
							Lower	Upper
TLMM	,021	,011	3,830	1	,050	1,021	1,000	1,043
RTdose	-,005	,023	,048	1	,826	,995	,950	1,042
