# Supplementary material for: Prognostic value of 11C-methionine volume-based PET parameters in IDH wild type glioblastoma
Source: PLoS One. 2022 Feb 25;17(2):e0264387. doi: 10.1371/journal.pone.0264387 (PMC8880430; doi:10.1371/journal.pone.0264387)
Supplement: S2 File — (PDF) [file pone.0264387.s002.pdf]

Supplement 2

S2a – Kaplan Meier curve for influence of age on survival

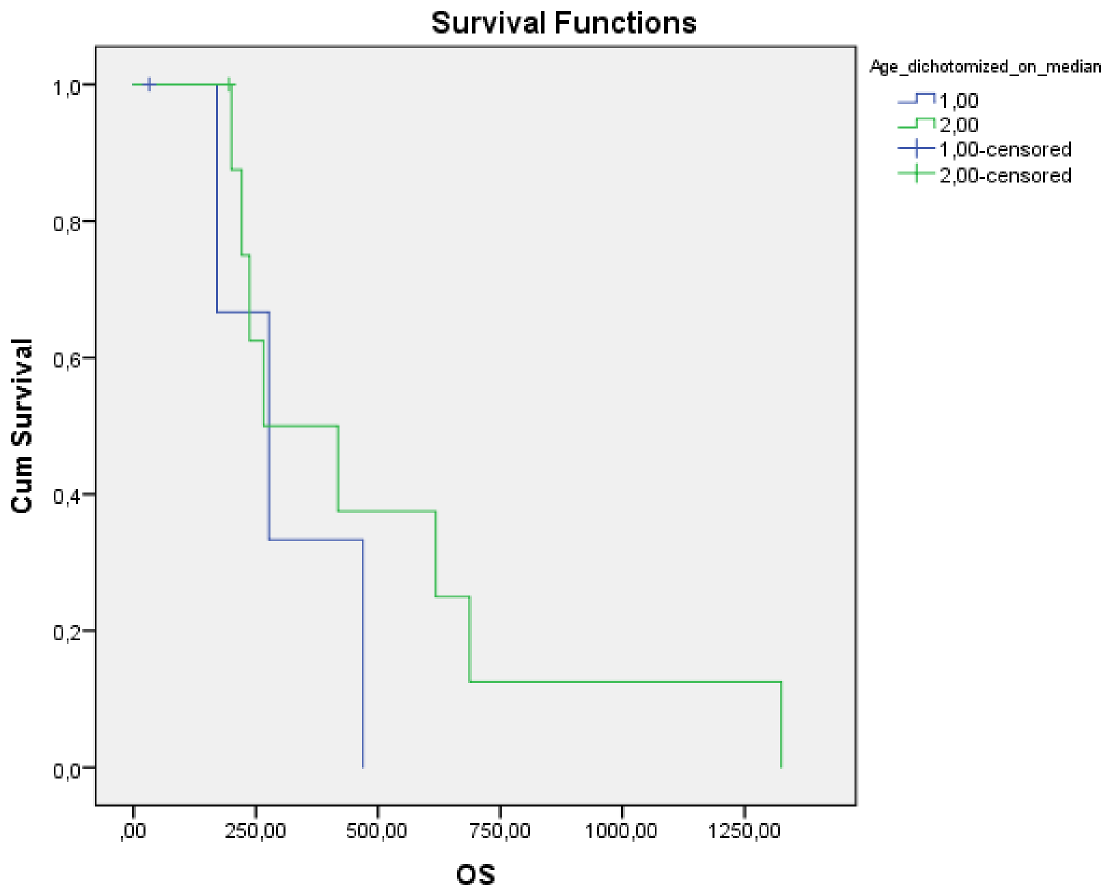

Data were dichotomized on median age of 57 years

S2b – Kaplan Meier curve for influence of age on survival

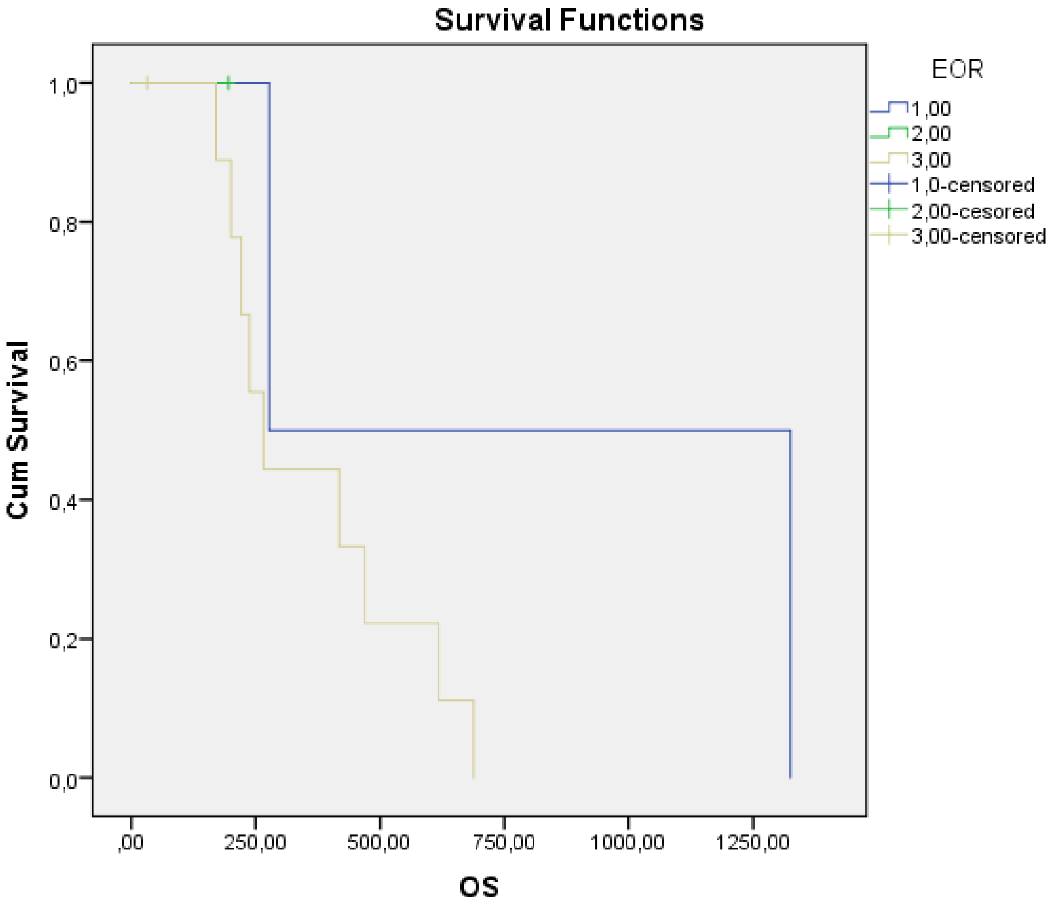

1 = biopsy; 2 = subtotal resection; 3 = gross-total resection
